# Verses

**Published:** 1907-04

**Authors:** 


### VERSES.

SAY.

If you are blue
  Cheer up!
If you are down
  Get up!

—Birmingham Age Herald.

Here's to the chaperone,
  May she learn from Cupid
Just enough blindness
  To be sweetly stupid.

STRIFE OF RESEARCH.

"Not a truth has to art
  Or to science been given,
  But brows have ached for it,
And souls toiled and striven."

—Exchange.

PEDIGREE.

The pedigree of honey
Does not concern the bee;
A clover, any time, to him
Is aristocracy.

—Emily Dickinson.

SAY IT.

When you've got a thing to say,
Say it! Don't take half a day.
When your tale's got little in it,
Crowd the whole thing in a minute!
Life is short—a fleeting vapor—
Don't you fill the whole blamed paper
With a tale, which, at a pinch,
Could be cornered in an inch!
Boil her down until she shimmers;
Polish her until she glimmers;
When you've got a thing to say
Say it! Don't take half a day!

—S. S. Times

THE JIM CROW CAR.

Rich folks ride een de parler kyar,
Een common coach, pore fokes go,
De nigger be hit near ur far,
Hab ter jurn een de Jim Crow.

Dem rowdy coons got no sense,
Dey brung dere color berry low,
Dey loss dere chance, and eber sense,
We hab ter go een de Jim Crow.

Wake up brudder get clean and mum,
Ef yer wanter stan long een de row,
Fer wite fokes, long es yer is bum
Gwine ter keep yer een de Jim Crow.

—B. H. Teague.

